# CYP2C19 Genotype, *CagA* Genotype and Antibiotic Resistant Strain of *Helicobacter pylori* Infection

**DOI:** 10.31557/APJCP.2019.20.4.1243

**Published:** 2019

**Authors:** Jeerayuth Auttajaroon, Peranart Chotivitayatarakorn, Yoshio Yamaoka, Ratha-Korn Vilaichone

**Affiliations:** 1 *Gastroenterology Unit, Department of Medicine, Faculty of Medicine, Thammasat University Hospital, *; 2 *National Gastric Cancer and Gastrointestinal Diseases Research Center (NGGC), Department of Medicine,*; 3 *Chulabhorn International College of Medicine (CICM) at Thammasat University, Pathumthani, Thailand, *; 4 *Department of Environmental and Preventive Medicine, Faculty of Medicine, Oita University, Oita, Japan.*

**Keywords:** CYP2C19 genotype, antibiotic resistant strain, Helicobacter pylori

## Abstract

**Background:**

*H. pylori *is a class I carcinogen and major cause of gastric cancer. Few previous studies reported relationship between *H. pylori *infection, CYP2C19 genotype and functional dyspepsia (FD) subtype. The aim of this study was to determine relationship between CYP2C19 genotype and FD subtype patients(host factor) with antibiotic resistant strains of *H. pylori* infection and *CagA* genotype(bacterial factor).

**Methods:**

FD patients who were investigated with gastroscopy at Thammasat University Hospital, Thailand during March 2017-November 2017 were enrolled. Two antral gastric biopsies were obtained for rapid urease test, E-test and cultures. *CagA* genotypes (*CagA1a* and *CagA2a*) were determined by PCR and CYP2C19 genotype was determined by PCR-RFLP. FD patients were categorized as epigastric pain syndrome(EPS) and postprandial distress syndrome (PDS).

**Results:**

93 FD patients with *H. pylori *infection were enrolled (37 male, 56 female, mean age 54.5 years). There were 33 patients with EPS and 60 patients with PDS. CYP2C19 genotype revealed 55.9% rapid metabolizer (RM), 40.9% intermediate metabolizer (IM) and 3.2% poor metabolizer (PM) genotypes. Antibiotics susceptibility tests demonstrated 62.8% resistant to metronidazole, 12.9% resistant to clarithromycin and 27.1% resistant to fluoroquinolone. *CagA 1a* and *CagA 2*a were demonstrated in 6 patients(11.5%) and 46 patients(88.5%). *CagA2a* genotype was more prevalent in PDS than EPS patients (94.3%vs.76.5%; P =0.08) without significance. In intermediate metabolizer (IM), *CagA2a* genotype was significant higher in PDS than EPS(100% vs.25%; P=0.004).

**Conclusions:**

PDS, CYP2C19 RM genotype and *CagA 2a *gene of *H. pylori* infection were the predominant type of FD in Thailand. Metronidazole remain the most common antibiotic resistant strain of *H. pylori* infection in FD patients. PDS (host factor) was significantly related to *CagA2a* genotype (bacterial factors) only in patients with intermediate metabolizer. Appropriate dose of proton pump inhibitor (PPI) and correct regimens for *H. pylori* eradication in FD patients should be consider to improve clinical outcomes.

## Introduction

*H. pylori* causes chronic gastric inflammation and lead to peptic ulcer disease (PUD), mucosa associated lymphoid tissue (MALT) lymphoma and gastric cancer (Marshall and Warren, 1984; Li et al., 2014; Miftahussurur et al., 2017, Vilaichone et al, 2017; Vilaichone et al., 2018). The International Agency for Research in Cancer classified *H. pylori* as a type I carcinogen and previous studies has found that *H. pylori* treatment could be reduced the incidence of gastric cancer (Vilaichone et al., 2011; Abebaw et al., 2014; Ford et al., 2014). *CagA* is a highly immunogenic protein encoded by the *CagA* gene. *CagA*-positive strains of *H. pylori *were associated with a higher inflammation and increased risk of worse outcomes than with *CagA*-negative strains. *CagA* genotype can be divided into *CagA 1a* and *CagA 2a*. *CagA 1a* strain of *H. pylori *demonstrated more virulence and associated with more gastric inflammation due to activation of proinflammatory cytokines eg. IL-1B, IL-6 and IL-8 (Yamaoka, 2010; Vilaichone et al., 2011)

Pharmacogenetic polymorphisms are long known affecting biotransformation and clinical outcome. The clinical importance of these variants depends on allele-frequency and the effect size of the clinical outcome parameters. The proton pump inhibitor (PPI) drugs are affected by genetic polymorphisms of the cytochrome P450 drug metabolizing enzyme CYP2C19. The outcome of CYP2C19 genotype testing were demonstrated as: rapid metabolizer (RM), intermediate metabolizer (IM) or poor metabolizer (PM). CYP2C19 genotypes have effect on pharmacokinetics and pharmacodynamics of PPI which involved in changing gastric pH and effectiveness of *H. pylori* eradication (Phiphatpatthamaamphan et al., 2016) 

Currently, *H. pylori* resistance rates to antibiotics are becoming problem in most parts of the world. *H. pylori *antibiotic resistance caused eradication rates have been declining while the prevalence of antibiotic resistance rates have been increasing (Malfertheiner et al., 2017) In Thailand, Antibiotic resistance was presented in 50.3% including amoxicillin 5.2%, tetracycline 1.7%, clarithromycin 3.7%, metronidazole 36%, ciprofloxacin 7.7%, Levofloxacin 7.2% and multi-drugs in 4.2% (Vilaichone et al., 2013). The aim of this study was to determine relationship between CYP2C19 genotype and FD subtype of patients (host factor) with antibiotic resistant strains of *H. pylori* infection and *CagA* genotype (bacterial factor).

## Materials and Methods

*Patients*

Eligible patients’ age between 18-70 years have been evaluated by gastroscopic examination at Thammasat University Hospital, Pathumthani, Thailand between March 2017 and November 2017. After endoscopy, those with diagnosis of functional dyspepsia (FD), which was established during gastroscopy with normal finding or mild gastritis, were considered entering in this study. FD patients were diagnosed by Rome IV diagnostic criteria (Stanghellini et al., 2016) and categorized as epigastric pain syndrome (EPS) and postprandial distress syndrome (PDS). Exclusion criteria included patients with (1) a history of previous *H. pylori* eradication, (2) on anticoagulants drugs (3) previous gastric surgery, (4) administration of antibiotics, bismuth and PPI drugs in the prior 4 weeks, (5) pregnancy and breast-feeding woman, (6) allergy to one of the study medication, and (7) other serious comorbidities (such as major systemic diseases, cardiovascular disease, malignancies). All patients provided written informed consent.

**Table 1:** Baseline Demographic Data of All Patients

Characteristic data	Frequency
Male : female	37:56
Mean age	54.5
Type of FD	
Epigastric pain syndrome	33 (35.5%)
Post prandial distress syndrome	60 (64.5%)
Antibiotic susceptibility test	
Metronidazole (M) resistance	27/43(62.8%)
Clarithromycin (C) resistance	9/70 (12.9%)
Fluoroquinolone (F) resistance	19/70 (27.1%)
M+C resistance	1/70 (1.4%)
M+F resistance	3/70 (4.2%)
C+F resistance	2/70 (2.8%)
CYP2C19 genotype	
Rapid metabolizer(RM)	52/93 (55.9%)
Intermediate metabolizer(IM)	38/93 (40.9%)
Poor metabolizer(PM)	3/93 (3.2%)
*CagA* genotype (N=52)	
* CagA 1a*	6/52 (11.5%)
* CagA 2a*	46/52 (88.5%)

**Table 2:** CYP2C19 Genotype and Antibiotic Susceptibility test of *H. pylori* in FD Patient

Factor		EPS (N=33)	PDS (N=60)	P-value 0.82
Sex				
	Female	19 (57.6%)	37 (61.7%)	
	Male	14 (42.4%)	23 (38.3%)	
Antibiotic susceptibility test				
	Metronidazole (M)	11/15 (73.3%)	16/28 (57.1%)	0.16
resistance*				
	Clarithromycin (C)	2/25 (8%)	7/45 (15.6%)	0.2
resistance**				
	Fluoroquinolone (F)	9/25 (36%)	10/45 (22.2%)	0.11
resistance**				
	M+C resistance**	0/25 (0%)	1/44 (2.3%)	0.32
	M+F resistance**	1/25 (4%)	2/45 (4.4%)	0.48
	C+F resistance**	1/25 (4%)	1/45 (2.2%)	0.35
CYP2C19 genotype				
	Rapid metabolizer(RM)	22/33 (66.7%)	30/60 (50%)	0.06
	Intermediate	10/33 (30.3%)	28/60 (46.7%)	0.06
metabolizer(IM)				
	Poor metabolizer(PM)	1/33 (3.0%)	2/60 (3.3%)	0.48
*CagA* genotype***		17/17 (100%)	35/35 (100.0%)	
	*CagA 1a*	4/17 (23.5%)	2/35 (5.7%)	0.08
	*CagA 2a*	13/17 (76.5%)	33/35 (94.3%)	

*N, 43; ** N, 70; *** N, 52

**Table 3:** Antibiotic Susceptibility Test of *H. pylori* and CYP2C19 Genotype

Antibiotic susceptibility test	RM (N=52)	IM (N=38)	PM (N=3)
Metronidazole (M) resistance	15/23(65.2%)	10/18(55.6%)	2/2(100%)
Clarithromycin (C) resistance	7/42(16.7%)	2/25(8%)	0/3(0%)
Fluoroquinolone (F) resistance	12/42 (28.6%)	6/25(24%)	1/3(33.3%)
M+C resistance	1/42 (2.4%)	0/25(0%)	0/3(0%)
M+F resistance	2/42 (4.8%)	0/25(0%)	1/3(33.3%)
C+F resistance	1/42 (2.4%)	1/25(4%)	0/3(0%)

All P value > 0.05

**Table 4:** Subgroup Analysis of *CagA* Genotype and FD Patient in Each CYP2C19 Genotype

CYP2C19 genotype and *CagA* genotype	EPS ( N=17)	PDS ( N=35)	P-valve
Rapid metabolizer(RM)			
*CagA*	12/12 (100%)	18/18 (100%)	
* CagA1a*	1/12 (8.3%)	1/18 (5.6%)	1.00
* CagA2a*	11/12 (91.7%)	17/18 (94.4%)	1.00
Intermediate metabolizer(IM)			
*CagA*	4/4 (100%)	16/16 (100%)	
* CagA1a*	3/4 (75%)	0	0.004
* CagA2a*	1/4 (25%)	16/16 (100%)	
Poor metabolizer(PM)			
*CagA*	1/1 (100%)	1/1 ( 100%)	
* CagA1a*	0	1/1 ( 100%)	1.00
* CagA2a*	1/1 ( 100%)	0	1.00

During the endoscopy, 2 biopsy samples from gastric antrum and body were obtained for rapid urease test, *H. pylori* culture and histological examination. *H. pylori *culture were obtained by inoculating the specimens on nonselective media (Columbia agar supplemented with 7% sheep blood) under a microaerophilic atmosphere for at least 7 days. The colonies were identified by gram straining, oxidase, catalase and urease production. Susceptibility for metronidazole was tested by Epsilometer test (E-test) or GenoType®HelicoDR.

*H. pylori CagA genotyping*

Chromosomal DNA was selected from plate cultures expanded from each single *H. pylori* colony using the QIAamp Tissue kit (QIAGEN Inc. Santa Clarita, CA, USA) according to the manufacturer’s instructions. Polymerase chain reaction (PCR) amplification was done for 35 cycles consisting of 1 min at 95°C, 1 min at 52°C and 1 min at 72°C. Final cycle included a 7-min extension step to confirm full extension of PCR products. *CagA* genotypes (*CagA1a* and *CagA2a*) were also determined by technique of PCR.

*CYP2C19 genotype*

DNA was extracted from gastric tissue by using QIAamp Tissue kit (QIAGEN Inc. Santa Clarita, CA, USA) and forward to perform PCR-RFLP with gel electrophoresis in order to identify difference in the CYP2C19 genotypes. The results of CYP2C19 genotype testing were expressed as: rapid metabolizer (RM), intermediate metabolizer (IM) or poor metabolizer (PM).

*Statistical analysis*

The association between *FD*, *CagA* genes, antibiotic resistance and CYP2C19 genotype were evaluated by using chi-squared. Association between categorical variables were determined using odds ratio and 95% confidence interval (95% CI). The P values < 0.05 were considered to be statistically significant. All analyses were performed by using SPSS Statistics version 23.0 (IBM Corp., Armonk, NY). The study was conducted according to good clinical practice guidelines and was approved by our university ethics committee.

## Results

Total of 93 FD patients with *H. pylori *infection were enrolled (37 male, 56 female, mean age 54.5 years). There were 33 patients with EPS and 60 patients with PDS. CYP2C19 genotype was performed in all patients and revealed 55.9% RM, 40.9% IM, and 3.2% PM genotypes. Antibiotics susceptibility tests demonstrated 62.8% resistance to metronidazole, 12.9% resistant to Clarithromycin and 27.1% resistant to fluoroquinolone. *Cag A* genotype was performed in 52 patients. *CagA 1a *and 2a were demonstrated in 6 patients (11.5%) and 46 patients (88.5%), respectively as demonstrated in Table 1.

*CagA2a* genotype was more common in PDS than EPS patients (94.3% vs. 76.5%; P = 0.08), but without statistical significance. Both FD subtypes were not related antibiotics susceptibility tests and CYP2C19 genotype as demonstrated in Table 2. CYP2C19 genotype was not related antibiotics susceptibility tests and *CagA* genotype as demonstrated in Table 3. In each CYP2C19 genotype, antibiotic susceptibility test of *H. pylori* was not significantly associated with FD subtypes of patient. In subgroup analysis of patients with intermediate metabolizer (IM), *CagA2a *genotype was significantly higher in PDS than EPS (100% vs. 25%; P = 0.004) as demonstrated in Table 4.

## Discussion

The majority of FD patients in Asian countries were PDS subgroup (Kawamura et al., 2001; Vilaichone et al., 2011; Piriyapong et al., 2014). In this study, we also demonstrated that PDS was predominant in our population. In Thailand, metronidazole resistance was found to be as high as in 11-36%, whereas fluroquinolone and clarithromycin resistance were 7-29% and 4-5 %, respectively (Sahara et al., 2012; Chotivitayatarakorn et al., 2017). In our study, antibiotics susceptibility tests demonstrated 62.8% resistant to metronidazole, 12.9% resistant to Clarithromycin and 27.1% resistant to fluoroquinolone.

*CagA* is a highly immunogenic protein encoded by the *CagA* gene. *CagA* plays major roles in gastric inflammation and injury in association with activated inflammatory cells infiltration. *H. pylori* strains isolated from Western and East Asian countries could be easily distinguished by PCR-based *CagA 5’* and* 3’* region genotyping, and named the East Asian type *H. pylori* strains as *CagA 1a *type and the Western type *H. pylori* strains as *CagA 2a* type. East-Asian type *CagA* is considered to be more toxic than its Western homologues and more strongly associated with severe clinical outcomes, including gastric cancer. (Sahara et al., 2012; Uchida et al., 2015) Recent study, prevalence of *CagA 1a* genotype was demonstrated in 11.6-24.6% in Thailand. (Vilaichone et al., 2011; Uchida et al., 2015) In our study, prevalence of *CagA 1a* genotype was demonstrated in 11.5%. Prevalence of *CagA 1a* genotype was lower than other Asian country. Also, the incidence of gastric cancer in Thailand was lower than other Asian populations.

Functional dyspepsia (FD) is common disease. The Rome IV Consensus proposed the subdivision of FD into two subgroups: the epigastric pain syndrome (EPS) and the postprandial distress syndrome (PDS). The pathophysiology is complex and multifactorial. The relationship between *H. pylori* infection and FD remains unclear, but there is evidence showing benefit of eradicating *H. pylori* in patients with chronic dyspepsia, with number needed to treat of 14. Cost-effectiveness analyses suggested that *H. pylori* eradication is the most cost-effective treatment for infected dyspeptic patients compared with alternative therapies that need to be taken long-term (Stanghellini et al., 2016). The term *H. pylori*-associated dyspepsia is recently introduced and defined as dyspepsia with long-term sustained remission of symptom after successful eradication of *H. pylori* (Sugano et al., 2015; Suzuki and Mori, 2015). Previous study showed that the prevalence of Cag A positive strains was not significant in PDS patients in Thailand (Vilaichone et al., 2011). Recent study from Taiwan reported association between *H. pylori* infection and FD patients with only pure PDS, but not with pure EPS or overlap syndrome. There was more prevalence of Cag A positive strains in PDS patients (Fang et al., 2015). In this study, we found equal prevalence of *CagA* positive *H. pylori* infection in PDS and EPS patients, since all patients in our study are *CagA* positive. *CagA 2a* were more prevalent in PDS compared with EPS patients without statistical significance, but *CagA 2a* were significantly more common in PDS in subgroup analysis of patients with IM. However, it should be noted that the magnitude of association between Cag A 2a positive strains and PDS symptom from subgroup analysis, even with statistical significant, could be resulted from unmeasured confounders. A further study is warranted to confirm the relationship between *CagA* genotype and FD in Asian populations.

In conclusions, PDS, CYP2C19 RM genotype and *CagA 2a* gene of *H. pylori* infection were the predominant type of FD in Thailand. Metronidazole resistance remain the most common antibiotic resistant strain of *H. pylori *infection in FD patients followed by fluoroquinolone resistant strain. PDS (host factor) was significantly related to *CagA2a* genotype (bacterial factors) only in patients with intermediate metabolizer. However, there were no association between FD subtype and CYP2C19 genotype or antibiotic resistance pattern of *H. pylori* infection in Thailand. Host and bacterial factors play important roles in pathogenesis of FD patients and might help to management for better outcomes in this particular disease. Appropriate dose of proton pump inhibitor (PPI) and correct regimens for *H. pylori* eradication in FD patients should be consider to improve clinical outcomes.
